# Scaffold Affinity Tunes Biomolecular Condensate Function

**DOI:** 10.64898/2026.08.30.748146

**Published:** 2026-09-01

**Authors:** Andres Reyna, Madyson O. Briggs, Alexander F. Russell, Tien M. Phan, Ryan J. Wang, Reuben Allen, Thomas R. Hinds, Ning Zheng, Jeetain Mittal, Champak Chatterjee

## Abstract

Biomolecular condensates (BMCs) organize cellular biochemistry by concentrating selected molecules into dynamic membrane-free compartments. Yet the molecular parameters that determine not only whether condensates form, but also how they behave and what they do, remain poorly defined. Here we show that scaffold binding affinity (K _d_ ) is a quantitative determinant of condensate phase behavior, internal dynamics and biochemical output. Using a modular SUMO-SIM system in which scaffold valency was held constant while binding affinity was systematically varied, we found that affinity governs the phase boundary, resistance to chemical perturbation, and molecular mobility of condensates in vitro and in human cells. In multicomponent mixtures, the highest-affinity scaffold dominated dense-phase composition and dynamics, revealing a hierarchical rule for condensate organization. Finally, affinity-dependent changes in condensate dynamics translated into tunable enzyme activity, establishing binding energetics as an engineerable parameter for programming condensate biochemistry.

## Full Text Availability

The license terms selected by the author(s) for this preprint version do not permit archiving in PMC. The full text is available from the preprint server.

